# Protective efficacy of folic acid and vitamin B12 against nicotine-induced toxicity in pancreatic islets of the rat

**DOI:** 10.1515/intox-2015-0016

**Published:** 2015-06

**Authors:** Ankita Bhattacharjee, Shilpi Kumari Prasad, Swagata Pal, Bithin Maji, Alak Kumar Syamal, Arnab Banerjee, Sandip Mukherjee

**Affiliations:** 1 Department of Physiology, Serampore College, Serampore, Hooghly – 712201, West Bengal, India; 2 Department of Physiology, Yogoda Satsanga Palpara Mahavidyalaya, Palpara, Purba Midnapore, West Bengal – 721 458, India; 3 Department of Physiology, Hooghly Mohsin College, Hooghly, West Bengal, India

**Keywords:** nicotine, islet cell, oxidative stress, folic acid, vitamin B12

## Abstract

Although cigarette smoking is associated with insulin resistance and an increased risk for type 2 diabetes, few studies have examined the effect of nicotine on the adult endocrine pancreas. In this study, male Wister rats were treated with nicotine (3 mg/kg body weight/ day) with or without supplementation of folic acid (36 μg/kg body weight/day) or vitamin B12 (0.63 μg/kg body weight/day) alone or in combination. Fasting blood glucose, insulin and HBA1C level and different oxidative and anti-oxidative stress parameters were measured and pancreatic tissue sections were stained with eosin-haematoxylene. Data were analysed by nonparametric statistics. The results revealed that nicotine induced prediabetes condition with subsequent damage to pancreatic islets in rats. Nicotine also caused oxidative stress in pancreatic tissue as evidenced by increased nitric oxide and malondialdehyde level and decreased superoxide dismutase, catalase and reduced glutathione level. Compared to vitamin B12 supplementation, folic acid blunted the nicotine-induced toxicity in pancreatic islets with higher efficacy. Further, folic acid and vitamin B12 in combination were able to confer significant protection on pancreatic islets against nicotine induced toxicity. These results suggest that supplementation of folic acid and vitamin B12 in combination may be a possible strategy of detoxification against nicotine-induced toxicity in pancreatic islets of the rat.

## Introduction

Cigarette smoking and type 2 diabetes are major public health burdens. Both are risk factors for cardiovascular disease and their co-occurrence has a dramatic impact on the absolute risk of mortality. Earlier cigarette smoking was reported to be associated with an increased risk of developing type 2 diabetes (Willi *et al*., 2007). Throughout the world, smoking is one of the leading causes of preventable death, yet tobacco use is still extremely common. By 2015, tobacco is projected to be responsible for 10 percent of all deaths worldwide and to kill 50 percent more people than HIV/AIDS (Jiang *et al*., 2010).

Nicotine is an alkaloid extracted from dry leaves of *Nicotiana tabacum* (Karlsson & Ahrén, 1998) and millions of people worldwide are exposed to it through smoking cigarettes and also via insecticide inhalation. Nicotine is not a direct cause of most tobacco-related diseases, but it is highly addictive (Balfour, 2002) and is responsible for some deleterious effects of smoking (McPhail *et al*., 1998; Singh *et al*., 2000). Reports indicate that nicotine affects a variety of cellular processes ranging from altered gene expression (Zhang *et al*., 2001) to secretion of hormones (Sano *et al*., 1999) and modulation of enzymatic activity (Yildiz *et al*., 1999)

Epidemiological studies have demonstrated a trend to higher serum glucose (Huang *et al*., 2007) and an increased prevalence of diabetes (Montgomery & Ekbom, 2002) in children born to smoking women. The early expression of nicotinic cholinergic receptors (nAChR) subunits alpha2-alpha4, alpha6, alpha7 and beta2-beta4 in the rat pancreas (Bruin *et al*., 2008b) yielded the hypothesis of a primary deleterious effect of prenatal nicotine exposure on the development of this organ. In fact, nicotine crosses the placental barrier and is present in the foetal circulation and amniotic fluid at higher levels than in the maternal circulation (Andres & Day, 2000). All these morphological and molecular alterations logically lead to altered physiological control of glucose homeostasis in animal models with early exposure to nicotine. Although an *in vitro* study with rat and human islets showed that acute or 48-hour exposure to nicotine might moderately inhibit insulin release (Yoshikawa *et al*., 2005), the effect of smoking on pancreatic insulin secretion is still controversial.

Exposure to nicotine has been reported to induce systematic oxidative stress and to disrupt the endogenous antioxidant defense mechanisms by down-regulating catalase (CAT) and superoxide dismutase (SOD) (Zaghloul *et al*., 2013). It is well known that cigarette smoke is the most common oxidant stress in daily life, but it is still debatable whether nicotine is responsible for the effects, due to free radical generation associated with tobacco use. Studies *in vitro* and *in vivo* on rodent cells showed that exposure to nicotine produced oxidative tissue injuries in the Chinese hamster, rat, and mouse, often resulting in a depletion of glutathione content (Bhagwat *et al*., 1998). Furthermore, the plasma of smokers was found to exhibit increased products of lipid peroxidation (Kharb & Singh, 2000). Oxidative stress is considered to take part in the pathogenesis of various diseases including diabetes (Gul *et al*., 2000). Oxidative stress plays a permissive role in the process of apoptosis leading to cell destruction in many types of cell lineages (Zhao & Wang, 2012). At cellular level, oxidative stress-mediated β-cell apoptosis can result from an imbalance between reactive oxygen species (ROS) generation and their clearance by antioxidants. Islet β-cells express low levels of major antioxidants such as superoxide dismutase, catalase and glutathione peroxidase and are therefore particularly vulnerable to the detrimental effects of ROS mediated cellular injury compared to other cell types (Drews *et al*., 2010; Bast *et al*., 2002). Thus, nicotine-induced oxidative stress may play an important role in the development of diabetes in smokers. Moreover, smokers were reported to consume fewer green vegetables and fruits, which are rich in antioxidants, than do non-smokers of both sexes (Suleyman *et al*., 2002).

It is believed that the requirement for antioxidant nutrients depends on a person's exposure to endogenous and exogenous reactive oxygen species. Since cigarette smoking results in an increased cumulative exposure to reactive oxygen species from both sources, it would seem logical for cigarette smokers to have an increased requirement for antioxidant nutrients, both dietary and supplementary (Kelly, 2002). Folic acid acts as an antioxidant (Moens *et al*., 2008) and folate plays an important role in DNA synthesis, repair and methylation (Lamprecht & Lipkin, 2003). Furthermore, smokers tend to have lower levels of folic acid and vitamin B_12_ (Mansoor *et al*., 1997). In addition, vitamin B_12_ supplementation is beneficial for treating many inflammatory diseases and also provides protection in oxidative-stress-associated pathologies.

The aim of the present study was therefore to investigate the involvement of oxidative stress in nicotine-induced alteration in the functional status of the endocrine pancreas and to assess the efficacy of folic acid and vitamin B_12_ in preventing nicotine-induced damage in this organ.

## Materials and methods

### Chemicals and reagents

Nicotine hydrogen tartrate, folic acid, vitamin B_12_ were purchased from Sigma-Aldrich. Sulfanilamide, phosphoric acid, naphthyl ethylene diamine dihydrochloride (NEDH), thiobarbituric acid (TBA), trichloroacetic acid (TCA), xanthine, bovine serum albumin (BSA), nitroblue tetrazolium (NBT) and xanthine oxidase were purchased from Merck (Darmstadt, Germany). All other reagents were of analytical grade.

### Animal model

All animal experiments were performed according to the ethical guidelines suggested by the Institutional Animal Ethics Committee (IAEC) of Serampore College, Serampore, West Bengal, India. Male albino rats (Wister strain) weighing 110–125 g were used in all the experiments. The animals were maintained in an environmentally controlled animal house (temperature 24±3 °C) and in a 12 h light/dark schedule with free access to water supply.

### Experimental design

For experiments, the rats were randomly divided into five groups consisting of six rats each: Group A, control; Group B, nicotine-treated; Group C, nicotine + vitamin B_12_ supplemented; Group D, nicotine + folic acid supplemented; Group E, nicotine + folic acid + vitamin B_12_ supplemented. The animals of all groups were provided a control diet composed of 71% carbohydrate, 18% protein, 7% fat and 4% salt mixture (Chanda *et al*., 1996). The dose and the administration route of nicotine were used as reported earlier by Chattopadhyay and Chattopadhyay (Chattopadhyay & Chattopadhyay, 2008). Animals in Group B, C, D and E received an intraperitoneal injection of nicotine tartrate (dissolved in 0.9% physiological saline) at an effective dose of 3 mg/kg body weight for 21 days, administered daily at 16:00 h to avoid diurnal variation. The performed dilution assured that 1 ml of physiological saline contained the required dose of nicotine. Simultaneously, animals in the control subgroup received intraperitoneal injection of 1 ml physiological saline. Animals of Group C were orally treated with vitamin B_12_ (0.63 μg/kg body weight/day) and those of Group D with folic acid (36 μg/kg body weight/day) (Mukherjee *et al*., 2006). Animals of Groups E were orally supplemented with folic acid (36 μg/kg body weight/day) and vitamin B_12_ (0.63 μg/kg body weight/day) (Mukherjee *et al*., 2006). To overcome the impact of any altered food intake, animals of Group A were pair-fed with the experimental groups B, C, D and E.

### Blood sample collection and measurement of glucose

After the experimental period was over (21 days), the animals were kept in overnight fasting. The next morning, blood samples were collected from retro-orbital veins. Glucose oxidase enzyme kit (E. Merck, India) was used for estimation of blood glucose from all the samples.

### Determination of HbA_1C_

The collected blood samples were mixed with EDTA (ethylenediaminetetraacetic acid) (as per specification of the kit) and were used for estimation of glycated haemoglobin (HBA_1C_) following the ion exchange resin-based method.

### Serum and plasma preparation

After the collection of blood for fasting blood glucose, HBA_1C_ and OGTT, the animals of all the groups were anaesthetised with light ether anesthesia and sacrificed by cervical dislocation, which is one of the recommended physical methods of euthanasia. Blood samples were drawn from the heart and plasma was separated for insulin assay.

### Determination of insulin

Plasma insulin was measured by enzyme linked immunosorbent assay (ELISA) using the kit Cayman chemicals, USA. The intra assay variation was 4.9%. As the samples were run at a time, there was no inter-assay variation. The level of insulin in plasma was expressed in μIU/ml.

### Preparation of pancreatic tissue extract

The abdomen was opened and a small portion of the pancreas from the gastro-spleenic part was quickly removed and placed in a beaker containing ice-cold Tris-HCL buffer (pH 7.4). It was cut into small pieces with the help of scissors, homogenised immediately in a glass homogenising tube equipped with a Teflon pestle. The homogenate was processed according to the method of Mukherjee *et al*. (2006).

### Protein determination in crude extract

The total protein content was measured by the Lowry method using BSA as standard (Lowry *et al*., 1951)

### Determination of nitric oxide production (NO) and MDA level

The role of nitric oxide synthase (NOS) was indirectly assessed by estimating the amount of NO production. Nitric oxide decomposes rapidly in aerated solutions to form stable nitrite/nitrate products. In the study, nitrite accumulation was estimated by Griess reaction (Raso *et al*., 1999) and was used as an index of NO production. Pancreas homogenates (100 μL) were loaded into microtitre plate followed by addition of 100 μL Griess reagent (1% (w/v) sulfanilamide in 5% (v/v) phosphoric acid and 0.1% (w/v) naphthyl ethylene diamine dihydrochloride) and incubated at room temperature for 10 min. Later the absorbance was taken at 550 nm using ELISA Reader (Thermo Scientific, USA). The amount of nitrite in the sample (micromolar·mg protein) was calculated from a sodium nitrite standard curve.

Quantitative measurement of MDA was performed following the thiobarbituric acid (TBA) test (Wills, 1987). Pancreas homogenate (2 mL) was mixed with 1 mL of 20% (v/v) TCA and 1mL of 0.67% (v/v) TBA and then boiled for 10 min. After cooling, the mixture was filtered through Whatman filter paper and reading of the filtrate was done at 530 nm. The amount of MDA formed was quantitated with TBA and used as an index of lipid peroxidation. The results were expressed as nanomoles of MDA per milligram of protein using molar extinction coefficient (1.56×10^5^ cm^2^/mmol).

### Determination of superoxide dismutase

The nitroblue tetrazolium (NBT) method of Beauchamp and Fridovich (1971), which is based on the inhibition of NBT reduction by SOD, was used for the determination of SOD activities. Briefly, 2.5 mL of 0.05 mol sodium carbonate buffer (pH 10) was mixed with 0.1 mL of 3 mmol/L EDTA, 3 mmol/L xanthine, 1.5 mg/mL bovine serum albumin, 0.75 mmol/L NBT, and the serum and homogenates of pancreas containing SOD. The reaction was initiated by adding 0.1 mL of 56 mU/mL xanthine oxidase. After 30 min of incubation, the reaction was terminated by adding 6 mmol/L CuCl2 and was centrifuged at 350 g for 10 min. Absorbance of blue formazan was recorded at 560 nm and 25 °C. The relative absorbance was then converted into unit of SOD activity per mg protein, where one unit of SOD activity was equivalent to the quantity of SOD that caused a 50% reduction in the background rate of NBT reduction.

### Determination of catalase

Catalase activity was assessed according to the method of Aebi (1984) by following the decomposition of H_2_O_2_ at 240 nm. The difference in absorbance per unit time was used as a measure of CAT activity. The values were expressed as U/mg protein.

### Determination of glutathione level

GSH was determined in pancreas samples according to the method of Ellman (1959). Pancreas homogenates (20 μl) were mixed with 200 μl of PBS and 10 μl of 5,5’-dithiobis-2-nitrobenzoic acid (DTNB). After 15 min of incubation, absorbance was taken at 412 nm. Results were expressed as mM/mg protein (Ellman, 1959).

### Study of DNA damage

Pancreatic tissue DNA was isolated using the GenElute Mammalian Genomic DNA Miniprep Kit (Sigma Aldrich) and electrophoresed on a 1.2% agarose gel in the presence of 0.1 μg/ml ethidium bromide at 80 V for 2–4 h. DNA fragmentation was visualised by UV light, and gel was photographed (Huang *et al*., 2007).

### Preparation of permanent slides for histological studies

Permanent slides of the pancreas were prepared and stained with eosin-haematoxylin for histopathological evaluation. Pancreatic tissue from all groups of animals was selectively taken from the gastro-splenic portion and was Bouin’s-fixed. Paraffin blocks were prepared, and 4 to 5 μm-thin sections were cut with a rotary microtome and routine microscopic slides were prepared. Haematoxylin and eosin-stained slides were light microscopically (Carl Zeiss, Primostar model) examined for histological evaluation.

### Statistics

Data were expressed as Mean ± SE. Kruskal-Wallis non parametric one-way analysis of variance (ANOVA) test was performed to establish whether or not scores of different groups differed significantly and to test intergroup significant difference, Mann-Whitney U multiple comparison test was performed by using StatDirect Software (UK). Differences were considered significant at *p*<0.05.

## Results

**Figure 1:**
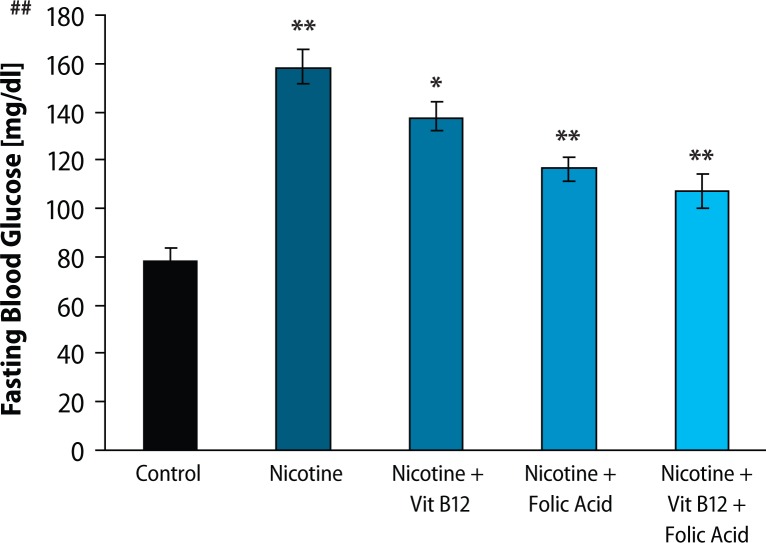
Effect of folic acid (36 μg/kg body weight/day), vitamin B_12_ (0.63 gg/kg body weight/day) and the combined effect of folic acid + vitamin B_12_ on nicotine (3mg/kg body weight/day) induced changes in fasting blood glucose level. Data expressed as Mean ± SE. Significance level based on Kruskal-Wallis test (*p*<0.001 ##). Control vs nicotine *p*<0.01**, nicotine vs nicotine + vitamin B12 *p*<0.05*, nicotine vs nicotine + folic acid *p*<0.01**, nicotine vs nicotine + folic acid + vitamin B12 *p*<0.01**.

**Figure 2:**
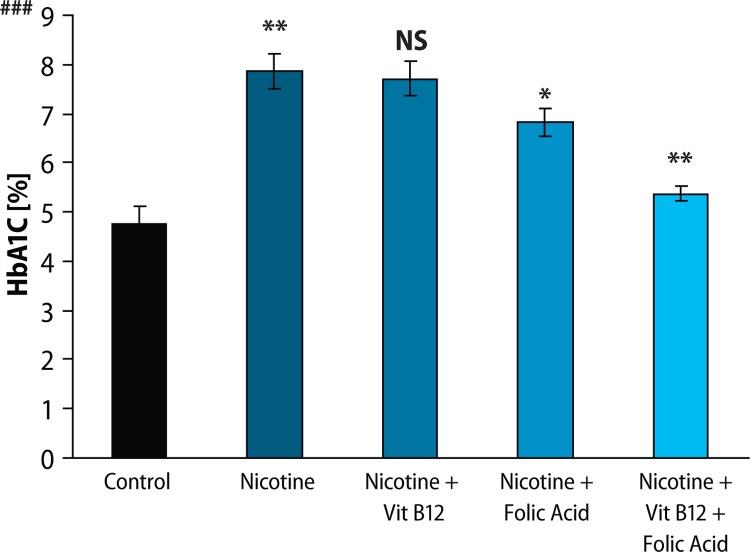
Effect of folic acid (36 pg/kg body weight/day), vitamin B_12_ (0.63 pg/kg body weight/day) and the combined effect of folic acid + vitamin B_12_ on nicotine (3mg/kg body weight/ day) induced changes in glycated haemoglobin level (HbA_1C_) level. Data expressed as Mean ± SE. Significance level based on Kruskal-Wallis test (*p*<0.001 ###). Control vs nicotine *p*<0.01**, nicotine vs nicotine + vitamin B12 NS, nicotine vs nicotine + folic acid *p*<0.05*, nicotine vs nicotine + folic acid + vitamin B12 *p*<0.01**. (NS-Not Significant).

**Figure 3:**
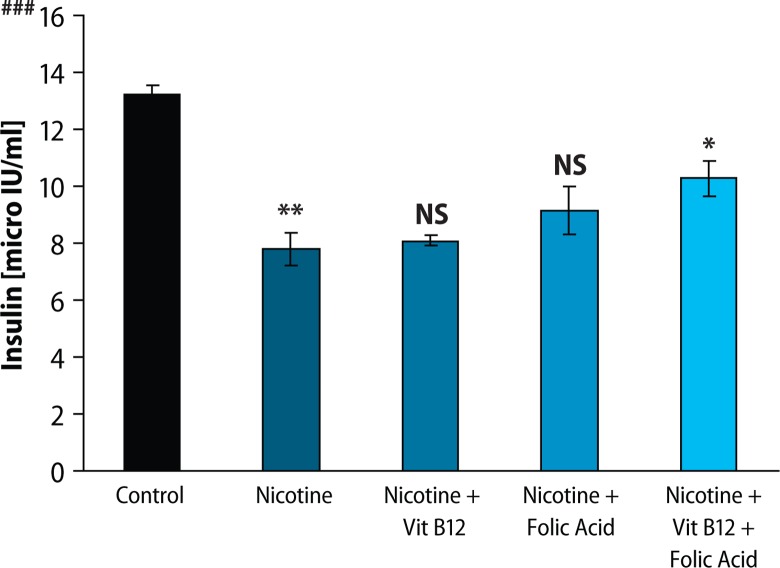
Effect of folic acid (36 μg/kg body weight/day), vitamin B_12_ (0.63 μg/kg body weight/day) and the combined effect of folic acid + vitamin B_12_ on nicotine (3 mg/kg body weight/day) induced changes of insulin level. Data expressed as Mean ± SE. Significance level based on Kruskal-Wallis test (*p*<0.001 ###). Control vs nicotine *p*<0.01**, nicotine vs nicotine + vitamin B12 NS, nicotine vs nicotine + folic acid NS, nicotine vs nicotine + folic acid + vitamin B12 *p*<0.05*. (NS-Not Significant).

**Figure 4:**
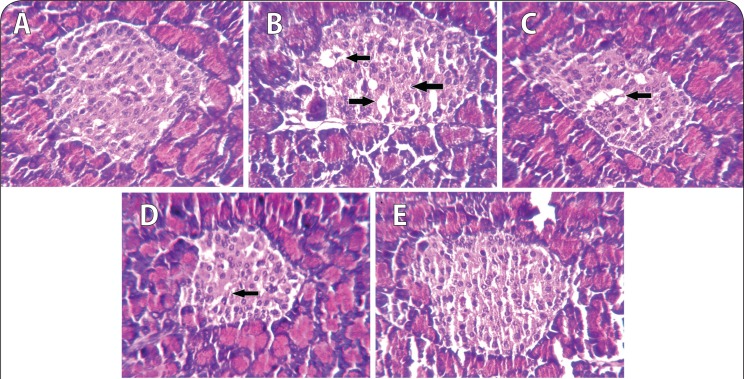
Representative photomicrograph of haematoxylene and eosine stained section (x40) showing morphology and population of cells in pancreatic islet of [A] control [B] nicotine (3 mg/kg body weight/day) [C] vitamin B_12_ (0.63 μg/kg body weight/day) [D] folic acid (36 μg/kg body weight/day) and [E] folic acid + vitamin B_12_ supplemented rat. In [A] no pathological changes were seen. Normal architecture of pancreatic islet and surrounding acini. Islet size and cell population normal. [B] Nicotine treated: typical characteristics of islet cell injury were present (marked by black arrow). Islet was shrunken; cell numbers were reduced. [C, D, E] marked recovery of cell injury changes by folic acid and vitamin B_12_ alone or in their combination.

Results of fasting blood glucose, HbA_1C_ level and plasma insulin level are presented in Figures 1–3, respectively. Both fasting blood glucose and HBA_1c_ level were found to increase significantly (*p*<0.01) in the nicotine treated group. Vitamin B_12_ supplementation significantly reduced (*p*<0.05) fasting blood glucose but was not able to recover the higher HBA_1c_ level caused by nicotine. Folic acid alone or in combination with vitamin B_12_ significantly altered the nicotine-induced changes in both fasting blood glucose and HBA_1c_ level. In addition, plasma insulin level was significantly (*p*<0.01) reduced in rats treated with nicotine and it was not ameliorated by the supplementation of vitamin B_12_ or folic acid alone. Further supplementation of folic acid in combination with vitamin B_12_ in nicotine-treated rats significantly prevented the nicotine-induced decrease in plasma insulin level. Eosin-haematoxylene stained pancreatic tissue section (Figure 4) revealed that nicotine caused destruction in islet architecture with increased vacuolisation. Combined supplementation of folic acid and vitamin B12 conferred protection against damaging effects of nicotine in pancreatic islets of the rats.

The results revealed that MDA and NO levels, hallmarks of lipid peroxidation and inflammatory response, were markedly increased (Table 1) in pancreatic tissue extract of the nicotine-treated group compared to the control group. Vitamin B_12_ alone was not able to provide protection against nicotine-induced increase in NO and MDA level. Yet folic acid alone or in combination with vitamin B_12_ treatment in other nicotine groups caused a marked decrease in both MDA level (*p*<0.01) and NO production (*p*<0.01).

**Table 1:** Table 1. Effect of Folic acid (36 μg/kg body weight/day), Vitamin B12 (0.63 μg/kg body weight/day) and the combined effect of Folic acid + Vitamin B12 (0.63 μg/kg body weight/day) on Nicotine (3.0 mg/kg body weight/day) induced changes in NO and MDA production, GSH level and activity of SOD and CAT in pancreatic tissue of rat.

Parameters	Control (Gr.A)[n=6]	Nicotine treated (Gr. B) [n=6]	Nicotine+ Vitamin B12 treated (Gr. C) [n=6]	Nicotine+ Folic acid treated (Gr. D) [n=6]	Nicotine+ Vitamin B12 +Folic acid treated (Gr. C) [n=6]	Sign. level*											
						Sign. level #	Gr. A vs Gr. B	Gr. B vs Gr. C	Gr. B vs Gr. D	Gr. B vs Gr. E	Gr. C vs Gr. D
NO Production [μM/mg/protein]	89.11±2.92	140.69±3.46	135.78±2.68	125.64±5.66	108.03±5.05	*p*<0.001	*p*<0.01	NS	*p*<0.05	*p*<0.01	NS
MDA production [nM/mg/ protein]	78.03±2.33	100.02±4.22	94.47±2.34	82.34±1.16	77.27±2.54	*p*<0.001	*p*<0.01	NS	*p*<0.01	*p*<0.01	*p*<0.01
SOD activity [U/mg protein]	184.45±3.48	145.21±2.35	152.83±1.80	169.91 ±2.74	177.04±3.81	*p*<0.001	*p*<0.01	*p*<0.05	*p*<0.01	*p*<0.01	*p*<0.01
CAT activity [U/mg/ protein]	1.28±0.08	0.69±0.13	0.87±0.17	1.02±0.04	1.20±0.09	*p*<0.05	*p*<0.01	NS	*p*<0.05	*p*<0.05	NS
GSH level [mM/mg protein]	59.58±1.52	40.33±1.03	42.99±1.10	44.17±1.43	54.32±1.15	*p*< 0.001	*p*<0.01	*p*<0.05	*p*<0.05	*p*<0.01	NS

Values are expressed as Mean±SE (n=6)

# Significance based on Kruskal Wallis test.

* Significance based on Mann-Whitney U multiple comparison test.

Chronic nicotine administration caused a significant decrease (*p*<0.01) in tissue GSH levels of the pancreas compared to the control group (Table 1). However, the nicotine-induced decrease in tissue GSH levels of the pancreas were reversed by supplementation with vitamin B_12_ alone (*p*<0.05) or folic acid alone (*p*<0.05) or folic acid in combination with vitamin B_12_ (*p*<0.01). In addition, after intoxication with nicotine, a decline in the activity of CAT (*p*<0.01) was observed in the nicotine-treated group when compared to the control group (Table 1). Vitamin B_12_ alone showed no protection against nicotine-induced decrease in CAT activity. But the CAT activity was restored towards normal in folic acid alone (*p*<0.05) and folic acid with vitamin B_12_ (*p*<0.01) treated groups. Furthermore, SOD activity was compromised significantly (*p*<0.01) in the nicotine-injected group, while vitamin B_12_, folic acid or folic acid with vitamin B_12_ supplementation in other nicotine-treated groups significantly (*p*<0.05, *p*<0.01, *p*<0.01, respectively) blunted the nicotine-induced changes in SOD activity.

**Figure 5:**
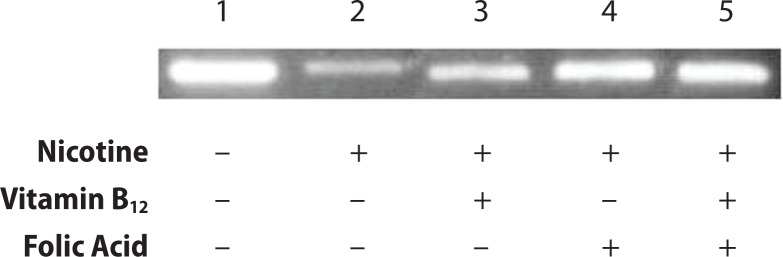
Effect of folic acid (36 μg/kg body weight/day), vitamin B_12_ (0.63 μg/kg body weight/day) and the combined effect of folic acid + vitamin B_12_ on nicotine (3 mg/kg body weight/day) induced genomic DNA damage in pancreatic tissue of rats. Lane 1: Control, Lane 2: nicotine treated, Lane 3: nicotine+vitamin B_12_, Lane 4: nicotine+folic acid, Lane 5: nicotine+folic acid+vitamin B_12_.

Figure 5 shows the effect of either folic acid or vitamin B_12_ or folic acid and vitamin B12 combined supplementation on islet cellular DNA damage of nicotine-treated rats. The results revealed that nicotine exposure caused an observable DNA damage (Lane 2) compared to control (Lane 1). Vitamin B_12_ supplementation alone to nicotine treated rats was not effective, whereas both folic acid and folic acid +vitamin B12 supplementation were found to be effective in reducing such DNA damage (Lane 4 and 5).

## Discussion

Nicotine in quantities similar to those in cigarette smoke can induce oxidative stress, as shown *in vitro* and *in vivo* (Solak *et al*., 2005). On the other hand, mounting evidence suggests that oxidative stress plays a role in the pathogenesis of diabetes mellitus and its complications (Brownlee, 2001). Hyperglycaemia increases oxidative stress, which contributes to the impairment of insulin action and insulin secretion. In addition, antioxidant mechanisms are diminished in diabetic patients, which may further augment oxidative stress (Rains & Jain, 2011). Several studies have addressed the possible participation of dietary antioxidants, such as vitamins, in ameliorating the diabetic state and retarding the development of diabetic complications (Sheikh-Ali *et al*., 2011). In this study we presented experimental evidence that nicotine causes oxidative stress and impairs the functional status of the endocrine pancreas, which can be effectively prevented by folic acid alone or in combination with vitamin B_12._

To investigate the effects of nicotine on *in vivo* pancreatic islet function, we monitored the changes in fasting blood glucose, HbA_1C_ level and plasma insulin level in nicotine exposed rats. Chronic exposure to nicotine, in our study, elevated fasting blood glucose level and HBA_1C_ level as compared to control animals. Insulin level, on the other hand, was significantly lowered in the nicotine-treated group, compared to control. Supplementation with vitamin B_12_ alone could not reverse the nicotine induced changes but folic acid alone or in combination with vitamin B_12_ significantly blunted the nicotine-induced changes in fasting blood glucose, HBA_1C_ and insulin level. Lower insulin level after nicotine treatment in this study was well in line with the earlier finding that either acute or chronic nicotine exposures could negatively affect insulin action to develop insulin resistance both in smokers before the onset of type 2 diabetes (DM 2) and in DM 2 patients (Xie *et al*., 2009). There are several lines of studies showing that nicotine can increase apoptosis of islet beta cells in nicotine exposed animal models (Bruin *et al*., 2008a; Bruin *et al*., 2008b). In this context, we can hypothesise that nicotine induces apoptosis of beta cells which may be responsible for the lower insulin level in this study, although we did not check apoptosis in the current setup. Further, haematoxylene-eosine stained sections of the pancreas exposed to nicotine showed loss of islet cells (Figure 4), which also supports this hypothesis. So, both nicotine-induced islet cell loss and decrease in insulin sensitivity may lead to hyperglycaemia and concomitant higher glycated haemoglobin (HbA_1C_), as observed in our study. Increased HbA_1C_, an objective index of chronic glycaemia, also further supports the earlier finding that smoking is associated with increased levels of glycated haemoglobin (HbA_1C_), (Sargeant *et al*., 2011). Although Mabley *et al*. (2002) reported that nicotine treatment reduced the incidence of type I diabetes in two animal models, on the basis of our results, we suggest that nicotine may be responsible for the disturbance in blood glucose homeostasis and islet cell damage. A further detailed study of the mechanisms of this disturbance is still required. Once nicotine-induced damage to beta cells begins and type 2 diabetes develops, several mechanisms come into play. High glucose itself causes oxidative stress in beta cells (Robertson *et al*., 2007) and leads to progressive loss of function. Supplementation with vitamin B_12_ alone showed no significant protection of islet cells exposed to nicotine but folic acid alone or in combination with vitamin B_12_ significantly blunted the nicotine induced changes in glucose homeostasis.

To further explore the involvement of oxidative stress in the mechanism underlying the toxicological effects induced by nicotine in the islets of the rat, we analysed nitric oxide production, lipid peroxidation, glutathione (GSH) content, as well as catalase (CAT) and superoxide dismutase (SOD) activity in pancreatic tissue homogenates. We observed increased NO production and lipid peroxidation in nicotine exposed animals (Table 1) as compared to controls, with concomitant decrease in GSH content and activities of SOD and CAT.

Nicotine, a major toxic component of cigarette smoke, is known to be a chemotactic for polymorphonuclear (PMN) leukocytes and enhances the responsiveness of PMN leukocytes to activated complement C5a, thus generating reactive oxygen species (Toklu *et al*., 2010). In the present study, marked elevation of NO levels in the pancreatic tissue extract of the nicotine group indicates enhanced generation of ROS. These ROS, in turn, are capable of initiating and promoting oxidative damage in the form of lipid peroxidation (Kovacic & Cooksy, 2005). High levels of NO production in pancreatic islets may negatively affect β-cell function. The presence of increased MDA level in pancreatic tissue extract of nicotine treated groups indicates the presence of enhanced lipid peroxidation, which is an autocatalytic mechanism leading to oxidative destruction of cellular membranes (Stark, 2005).

Disruption of the mitochondrial respiratory chain leading to leakage from the electron transport in cardiomyocytes of rabbits (Gvozdjakova *et al*., 1992), decrease in glutathione level in Chinese hamster ovary cells (Yildiz *et al*., 1999), and decreased activities of catalase and SOD in various tissues of the rat (Helen *et al*., 2000) were reported previously as proposed mechanisms by which nicotine produces oxidative stress. Further, addition of free radical scavenging enzymes SOD and CAT prevented nicotine-induced increase in lipid peroxidation in pancreatic tissue of the rat (Suleyman *et al*., 2002) and decrease in cellular glutathione level in Chinese hamster ovary cells (Yildiz *et al*., 1999). In agreement with the decreased SOD activity found in various rat tissues (Helen *et al*., 2000), decreased erythrocyte SOD activity would be involved in the nicotine-induced oxidative stress in the present study. Supplementation of folic acid alone or in combination with vitamin B_12_ significantly blunted the nicotine-induced alteration in both SOD and CAT level. But vitamin B_12_ alone was not effective in reversing the nicotine-induced decrease in CAT activity in the pancreatic tissue of the rat. Earlier reports revealed that nicotine-induced toxicity was coupled with GSH depletion, which is one of the essential compounds for maintaining cellular integrity (Mandrup-Poulsen, 2003). Nicotine exposure in the present study significantly depleted GSH stores in pancreatic tissue, indicating that GSH was used as an antioxidant for the detoxification of toxic oxygen metabolites, enhancing the susceptibility of the pancreatic islets to oxidative injury. On the other hand, combined supplementation of folic acid and vitamin B12 blunted nicotine-induced oxidative injury with a concomitant maintenance of GSH stores in the pancreas, implicating antioxidant action in improving tissue functions.

Since the results of the present study showed that oxidative stress was involved in the pathogenesis of nicotine-induced impairments in pancreatic islet cell functions, free radical ablation with antioxidant agents seems to be beneficial in preventing oxidant-induced tissue damage. Cigarette smoking is known to be associated with raised homocysteine levels (Reis *et al*., 2000). Smokers also tend to have lower levels of folic acid and vitamin B_12_ (Pagan *et al*., 2001). Both affect homocysteine levels, *i.e.* vitamin B_12_ as co-substrate and folic acid required for the enzymes controlling homocysteine metabolism (Muzawar & Patil, 2011). Further, folic acid has been well-defined as an effective free-radical scavenger and reported to inhibit lipid peroxidation. Antioxidant activity was reported to be more efficient when antioxidants were used in combination (Sahin *et al*., 2003).

In this study, supplementation of folic acid with or without vitamin B_12_ to nicotine treated rats was found to be successful in reversing the nicotine induced impairment in blood glucose homeostasis. These results were associated with increased islet beta cell mass and preserved islet architecture in nicotine treated rats supplemented with folic acid alone or in combination with vitamin B_12_. But vitamin B_12_ alone showed little or no protection against nicotine-induced damage in pancreatic islets in rats. A further study with some higher doses may be required in this aspect. In the present study, folic acid as a powerful antioxidant in combination with vitamin B_12_ inhibited NO production and MDA levels and potentiated SOD and CAT activity in pancreatic tissue extract of nicotine exposed rats implicating that tissue integrity is maintained by inhibiting the breakdown of membrane phospholipids by lipid peroxidation (Table 1). These results in our study are also well in line with earlier reports stating that oxidative stress plays a crucial role in inducing pancreatic islet beta cell injuries and that the pathogenesis of diabetes mellitus is probably a result of excessive levels of mitochondrial ROS production and the presence of fewer antioxidant enzymes in pancreatic beta cells (Drews *et al*., 2010).

Further, oxidative stress causes tissue damage in the pancreas and the extent of damage correlates with the loss of β-cell mass (Sakuraba *et al*., 2007). In the current study we assessed the extent of DNA damage in pancreatic tissue of nicotine treated rats and its protection, if any, by supplementation with vitamin B_12_ or folic acid or in combination of folic acid and vitamin B_12_. The results revealed that either folic acid or folic acid+vitamin B_12_ exert recovery potential and help prevent the nicotine induced DNA damage in pancreatic tissue. Yet vitamin B_12_ supplementation alone had no such ameliorating effect.

In conclusion, folic acid alone or combined with vitamin B_12_ prevented the nicotine mediated disturbance in glucose homeostasis and pancreatic islet cell damage, but vitamin B_12_ alone had no such preventive effect against nicotine toxicity in pancreatic tissue of the rat. It seems that alterations of antioxidant enzyme activities in response to nicotine-induced lipid peroxidation may be responsible for the effect of nicotine on the endocrine pancreas. The remarkable increase in GSH and activities of SOD and CAT in folic acid or folic acid+vitamin B_12_ supplemented groups might have contributed to the prevention of nicotine-induced oxidative stress and restoration of damaged islet cells and glucose homeostasis. The results of this study suggest that folic acid in combination with vitamin B_12_ can be used as nutritional supplement, especially by people who smoke, in order to prevent nicotine-induced oxidative stress and islet cell damage.
